# The Influence of the Global Gene Expression Shift on Downstream Analyses

**DOI:** 10.1371/journal.pone.0153903

**Published:** 2016-04-19

**Authors:** Qifeng Xu, Xuegong Zhang

**Affiliations:** 1 MOE Key Laboratory of Bioinformatics and Bioinformatics Division, TNLIST/Department of Automation, Tsinghua University, Beijing, China; 2 Department of Aircraft Spare Management, Air Force Logistic College, Xuzhou, Jiangsu, China; Jilin University, CHINA

## Abstract

The assumption that total abundance of RNAs in a cell is roughly the same in different cells is underlying most studies based on gene expression analyses. But experiments have shown that changes in the expression of some master regulators such as c-MYC can cause global shift in the expression of almost all genes in some cell types like cancers. Such shift will violate this assumption and can cause wrong or biased conclusions for standard data analysis practices, such as detection of differentially expressed (DE) genes and molecular classification of tumors based on gene expression. Most existing gene expression data were generated without considering this possibility, and are therefore at the risk of having produced unreliable results if such global shift effect exists in the data. To evaluate this risk, we conducted a systematic study on the possible influence of the global gene expression shift effect on differential expression analysis and on molecular classification analysis. We collected data with known global shift effect and also generated data to simulate different situations of the effect based on a wide collection of real gene expression data, and conducted comparative studies on representative existing methods. We observed that some DE analysis methods are more tolerant to the global shift while others are very sensitive to it. Classification accuracy is not sensitive to the shift and actually can benefit from it, but genes selected for the classification can be greatly affected.

## Introduction

Whole-genome gene expression analysis has become a major theme in many biological studies since the development of high-throughput genomic technologies like DNA microarrays and RNA sequencing [1–6]. There are already over 1,722,895 gene expression data samples in the NCBI Gene Expression Omnibus (GEO) public database [7] as of Feb, 2016.

All gene expression experiments with microarray [8] or RNA sequencing [9] must control the quantity of RNA molecules of each sample, and most experiments assume that the total amount of RNAs across cells are roughly the same. If this assumption is true, controlling the total abundance of RNA molecules of a sample is equivalent to controlling the total number of cells measured in the experiment. This is the base for all downstream analyses of the expression data. In 2012, several studies showed that the total RNA abundance of a cell with high levels of c-MYC expression can be two or three fold higher than those of cells with normal c-MYC expression [10–12]. Loven et al [12] discussed that common experimental methods using samples with similar amounts of total RNAs had relied on the incorrect assumption that cells produce similar levels of total RNAs. Such studies could draw wrong conclusions from gene expression experiments. For example, some up-regulated DE genes can be wrongly identified as down-regulated DE genes. They designed an experiment to show that the conventional pipeline of the major gene expression technologies failed to detect gene expression levels correctly, and they proposed that spiked-in controls should be used to avoid or rectify the influence of this type of global gene expression shift [12].

This is not a special rare case. Actually it has been known that c-MYC is a major master regulator that plays important roles in many processes like development and cancers [13, 14]. There have been more than 26,000 papers on it in PubMed. Besides the global gene expression shift that can be caused by c-MYC, other factors can also lead to unequal total expression per cell [15]. c-MYC and other master factors have been observed to be abnormally expressed in many cancers. Therefore, the massive existing data of cancer gene expression studies are more likely to be affected by the global gene expression shift. There have been many works on gene expression data normalization but none of them had taken into consideration of the possible global shift of gene expression levels between cells [16–19].

The data reported in Loven et al indicated that some up-regulated genes could be wrongly detected as down-regulated genes if the shift effect was not considered [12]. But the data were of a small scale and only from one particular study. It is largely unknown how much influence the global shift can have on a wide range of gene expression data for typical downstream analyses [20]. Therefore, we conducted a systematic study on this influence on two major types of downstream analyses: detection of differentially expressed genes [21, 22] and sample classification based on selected genes [23–28]. We analyzed a hypothetic model on the possible influence in the ideal setting, and designed experiments on Loven et al’s data with known shift effects as well as on data generated by simulating the global shift on 20 sets of gene expression data of various types. We adopted 3 representative methods for differential expression detection [29–31], two support vector machine (SVM)-based machine learning methods for gene selection and sample classification [27, 28], and compared their results on data with and without global gene expression shift. We observed that methods for detecting differential gene expression based on fold-change criteria are more robust to global expression shift than statistical-test-based methods. For sample classification, the two classes become more separable when global shift is present in one class, but the genes selected for the classification can be largely different with or without global shift.

## Problems and Methods

### Problems Analyses

The most widespread research question of gene expression analysis is the identification of changes in gene expression levels between the test group and the control group, e.g., comparing samples of a disease state to the normal state. The initial step of gene expression analysis experiment is to extract RNAs from tissues or cell samples we want to investigate, and many experiments control an equal weight of total RNAs in all samples of the test and control groups [8]. The cells of test sample are about as many as the cells of the control sample if the total amount of RNAs in experiment is roughly equal in each cell. The assumption that the total amount of RNAs of the cell is roughly equal is critical to the correct interpretation of a gene expression experiment. The unfair comparison of gene expression level between test and control group will arise if the assumption is violated.

The number of cells in the test group will be less than that of the control group if the amount RNAs of a cell in a test sample is greater than that in a control sample due to the global gene expression shift. We call such cases as the amplification effect for the convenience of discussion. On the other hand, the number of cells in test samples will be greater than that in control samples when the amount of RNAs of a cell in test samples is less than that of a cell in control samples. We call this case of global shift as the de-amplification effect. In a typical gene expression study, we actually don’t know whether there is amplification effect or de-amplification effect and all calculations on gene expressions take it for granted that signals of different samples were obtained from the same amount of cells.

Unequal numbers of cells in two groups can result in the wrong calculation of gene expression values. We can use a simple hypothetic toy example to intuitively study the influence of global shift. We assume there is a shift factor of 2.0 in the test sample compared with the control sample. That is, the total amount of RNAs in a cell of the test sample is twice as many as that in a cell of the control sample. Suppose we do two experiments: Experiment #1 controls the same total RNA amount for the two samples as that have been done in most microarray or RNA-seq experiments, and Experiment #2 controls the same number of cells for the two samples. Suppose 2000 cells are used in the control group in both experiments. Only 1000 cells of the test group will be used in Experiment #1, while Experiment #2 will use 2000 cells in the test group. Consider two genes A and B in the two experiments as illustrated in Table 1. Suppose the observed expression of them in the control sample are *X*_*A*_ and *X*_*B*_, respectively, in both experiments. If the observed expression of A and B in the test sample in the Experiment #2 are *Y*_*A*_ and *Y*_*B*_, respectively, their expression values will be 0.5*Y*_*A*_ and 0.5*Y*_*B*_ in Experiment #1. We can see that the ratios of gene expression between the two groups changed although their relative orders in each sample do not change. This can affect the judgment on the differential expression of genes: if the true per-cell expression of a gene is doubled in the test group, it’ll be detected as no-change in Experiment #1, and a gene with no-change per-cell expression will be detected as down-regulated in Experiment #1. This toy example was just about the comparison of two individual samples. When we consider two groups of samples, the change of the mean and variance of gene expression due to global shift will be more complicated. Therefore, we designed a series of experiments on real and simulation data to study the effect on the detection of differentially expressed genes as well as on sample classifications based on selected genes.

**Table 1:** A simple illustrative hypothetic example on the effect of global expression shift.

Experiment	#1		#2	
Group	Control	Test	Control	Test
Shift factor	1.0	2.0	1.0	2.0
The number of cell	2000	1000	2000	2000
Expression of gene A	*X*_*A*_	0.5*Y*_*A*_	*X*_*A*_	*Y*_*A*_
Expression of gene B	*X*_*B*_	0.5*Y*_*B*_	*X*_*B*_	*Y*_*B*_
Test-control ratio of gene A	0.5*Y*_*A*_/*X*_*A*_		*Y*_*A*_/*X*_*A*_	
Test-control ratio of gene B	0.5*Y*_*B*_/*X*_*B*_		*Y*_*B*_/*X*_*B*_	

### Loven et al's Data with Experimentally Verified Global Expression Shift

The data from Loven et al [12] include two samples of cells expressing a low level of c-MYC and two samples of cells expressing a high level of c-MYC. The gene expression data were obtained using the GeneChip^®^ PrimeView^™^ Human Gene Expression Array (with External spike-in RNAs). They used RNA spike-in controls to mark the number of cells in each sample, and validated that the samples with high c-MYC level have a global shift factor of about 2–3 comparing to the samples with low c-MYC level [12]. The true per-cell expression of genes can be obtained if the spike-in controls information is used in the normalization. If this information about cell numbers is not used, the estimate of per-cell expression of genes will be affected by the global shift. We separately detected up-regulated and down-regulated DE genes on the expression data with spike-in control information and on the expression data without using spike-in controls. We then compare the DE gene lists of the two experiments to check the overlap proportion. Fold-change and SAM were used as representations of methods for DE gene detection.

### Data with Simulated Global Shift

The data in Loven et al's work were of small scale. We designed a set of experiments on 20 sets of gene expression data of different sample sizes. All data sets were composed of two groups of samples for comparison. Since it is unknown whether there was global shift in the data, we artificially added simulated global shift to the data. That is, for each dataset, we generated a sister dataset by amplifying the expression of all genes in samples of one group (usually the disease group) by a factor following N(α, 0.1α) where α is the global shift factor. We experimented with α = 2, 4 and 8, and reported the results with α = 2 in this paper as it is close to the real situation observed in Loven et al’s data [12]. Observations on data with other shift factors were similar. With this setting, we simulated the situation that we have two versions of expression data in each study: one with global shift corrected and the other not corrected. We applied the same downstream analyses on the two sister datasets and compare the results. The discrepancy between the two results indicates the influence of global shift effect. We compared the overlap between lists of detected genes on the sister datasets with the same methods, and also compared classification accuracies on the sister datasets.

The databases are all gene expression data on various types of cancers downloaded from the GEO database [7]. Each dataset has a cancer group and a normal group. Table 2 gives the full list of the 20 datasets. The data were all obtained by Affymetrix microarray platforms, but they can represent the general situation of expression data with regard to the possible influence of global shift effects.

**Table 2:** Gene expression datasets used in the experiments.

Data ID	Cancer name	Paper information	Number of samples	Number of Cancer(Normal) samples	Number of Probeset
1	bladder	Dyrskjøt,L. et al. Cancer Res 2004	43	29(14)	22215
2	brain	Sun,L. et al. Cancer Cell 2006	61	38(23)	54613
3	cervical	Scotto,L. et al. Genes Chromosomes Cancer 2008	52	28(24)	22215
4	cervical	Zhai,Y. et al. Cancer Res 2007	31	21(10)	22215
5	colorectal	Sabates-Bellver,J. et al. Mol Cancer Res 2007	64	32(32)	54613
6	colorectal	Hong,Y. et al. Clin Exp Metastasis 2010	82	70(12)	54613
7	esophageal	Hu,N. et al. BMC Genomics 2010	34	17(17)	22215
8	esophageal	Su,H. et al. Clin Cancer Res 2011	106	53(53)	22215
9	esophageal	Su,H. et al. Clin Cancer Res 2011	102	51(51)	22477
10	Head neck	Kuriakose,MA. et al. Cell Mol Life Sci 2004	44	22(22)	12558
11	Head neck	Pyeon,D. et al. Cancer Res 2007	56	42(14)	54613
12	leukemia	Stirewalt,DL. et al. Genes Chromosomes Cancer 2008	64	26(38)	22215
13	lung	Landi,MT. et al. PLoS One 2008	107	58(49)	22215
14	lung	Spira,A. et al. Nat Med 2007	187	97(90)	22215
15	lung	Stearman,RS. et al. Am J Pathol 2005	39	20(19)	12558
16	lung	Su,LJ. et al. BMC Genomics 2007	54	27(27)	22215
17	pancreatic	Badea,Pancreas. et al. Hepatogastroenterology 2008	78	39(39)	54613
18	pancreatic	Pei,Pancreas. et al. Cancer Cell 2009	52	36(16)	54613
19	prostate	Yu,YP. et al. J Clin Oncol 2004	75	58(17)	12579
20	prostate	Wallace,TA. et al. Cancer Res 2008	87	69(18)	22215

For each of the gene expression datasets, we downloaded the expression data from GEO and generated a sister dataset as above illustrated. Then we took the following pre-processing procedures: We filtered genes with the maximum expression level in all samples less than 200, filtered genes with small variance between samples (the ratio of the maximum and minimum expression levels less than 3 fold, or the difference between the maximum and minimum expression levels less than 100), and set expression levels lower than 20 to 20. We adopted these preprocessing following most practices on microarray data analysis to avoid the possible complication of results caused by genes with very low expression or with little variations in their expression. But we also experimented with the original raw data and the results were consistent.

### Methods for Detecting Differentially Expressed Genes

We chose three commonly used methods for detecting differentially expressed genes: fold-change, t-test and SAM. Let *x*_*ij*_ and *y*_*ij*_ denote the expression levels of gene *i* in replicate *j* in the control and test samples, respectively, and ${\bar{x}}_{i}$ and ${\bar{y}}_{i}$ represent average expression levels of gene i in the control and test samples. The fold-change (ratio) for gene *i* is simply defined as ${\text{FC}}_{\text{i}}=\frac{{\bar{x}}_{i}}{{\bar{y}}_{i}}$. T-test is a basic statistic hypothesis testing method. We adopted the Welch's t-test [32] because it does not require the assumption of equal variances. Welch's t-test for gene *i* is defined as ${\text{T}}_{\text{i}}=\frac{{\bar{x}}_{i}-{\bar{y}}_{i}}{\sqrt{\frac{s_{1i}^{2}}{N_{1i}}+\frac{s_{2i}^{2}}{N_{2i}}}}$, where $s_{1i}^{2}$ and $s_{2i}^{2}$ are sample variances of the gene in the two groups, and *N*_1*i*_ and *N*_2*i*_ are sample sizes of two groups, respectively. SAM is a modified t-test method that added a constant to the standard deviation term in order to produce more stable results [31]. The SAM statistics is defined as ${\text{T}}_{\text{i}}=\frac{{\bar{x}}_{i}-{\bar{y}}_{i}}{s_{i}+s_{0}}$, where s_0_ is a constant. The SAM R package is used in our study and the parameters of the function SAM were set to the default value and the parameter resp.type was set to “two class unpaired”.

### Methods for Sample Classification and Gene Selection

Another type of downstream analysis is the classification of samples with machine learning methods based on a subset of genes selected from the expression data. There are two major strategies for this task. The filtering strategy adopts some criteria to select genes first and then build classification models based on the selected genes. A typical criterion for gene selection in this strategy is to select differentially expressed genes. Another strategy is the wrapper strategy, which integrates the selection step with the classification model. The influence of global shift on the filtering strategy will be largely decided by the influence on the detection of differentially expressed genes. Therefore, we focus on the wrapper strategy in studying the influence of global shift on sample classification and gene selection.

We chose two commonly used methods for sample classification and gene selection: R-SVM [27] and SVM-RFE [28]. Their basic ideas are very similar and the results were also close. So we present the results with R-SVM. Both methods are based on the support vector machine (SVM), which is a machine learning method that can perform well for classification of high-dimensional data with small sample sizes [27, 28]. The key idea of the SVM is to maximize the margin separating the two classes while minimizing the total classification errors [33].

R-SVM is a method uses linear SVM for classification and selecting subsets of relevant features (genes) according to their contribution in the classification [27, 34]. The selection was done in a recursive manner in multiple steps along a ladder of decreasing size of subsets, so that a subset of genes was selected from genes used in the previous step of SVM training. The contribution of a feature is evaluated by its differences between the mean of two classes multiplied by their weights in the trained SVM.

**Fig 1:**
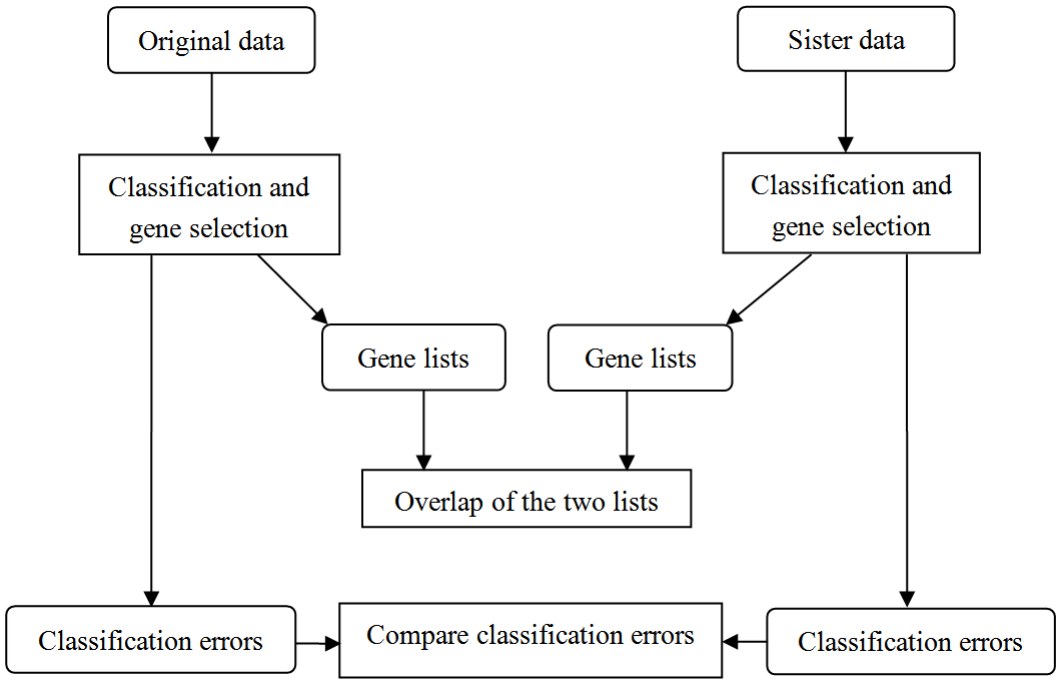
The flowchart of the classification and gene selection experiments on data with simulated global shift.

The stringent cross-validation scheme (CV2 as defined in [27]) is used to estimate the error rate of R-SVM at each level of feature selection. The samples to be tested in the validation step were left out at the beginning before any feature selection step. This avoids the possible over-fitting caused in feature selection caused by “information leak” due to the improper timing of cross-validation [27]. The detail of the method was described in [27]. We used R-SVM with C = 10 in the leave-one-out cross-validation and set the number of features to decrease by 50% at each level of feature selection along the ladder. We applied R-SVM on the original expression datasets and their sister data with simulated global expression shift, and compared the classification errors and the selected gene lists on each pair of datasets. Fig 1 shows the experiment diagram.

## Results and Discussion

### Results on Loven et al's Data

**Fig 2:**
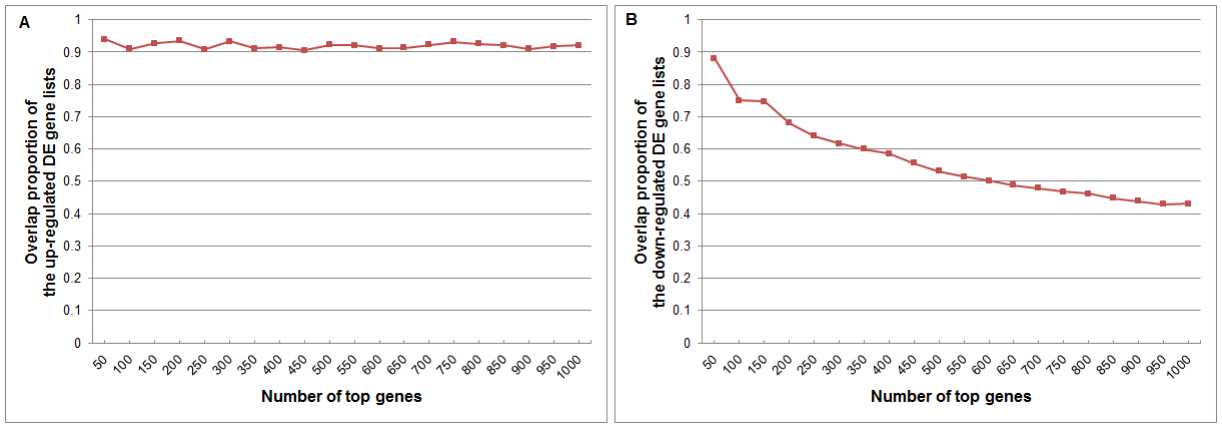
Overlap proportions of differentially expressed genes detected by fold-change from the data with corrected and uncorrected global shift effects on Loven et al’s data. (A) Up-regulated DE genes. (B) Down-regulated DE genes. The x-axis is the number of the top genes of the up-regulated DE gene lists or the down-regulated DE gene lists. The y-axis is the overlap proportions of the top genes.

We compared the overlap proportions of the top 50, 100, …, 1000 genes of the detected up-regulated DE gene lists and the down-regulated DE gene lists obtained from the data corrected with spike-in-controls and un-corrected data. The results of fold-change are shown in Fig 2. We can see that the overlap proportion of the up-regulated genes is always high, and the overlap proportion of the down-regulated genes is high for the top few genes, but decreases rapidly when we go down in the list. This is consistent with the understanding that for up-regulated genes, global shift will make them more up-regulated and therefore won’t change the order much. But for down-regulated genes, many of them are actually also up-regulated and the seemingly down-regulation are due to the improper normalization. Therefore, correcting the global shift will cause big change in the list of down-regulated genes.

**Fig 3:**
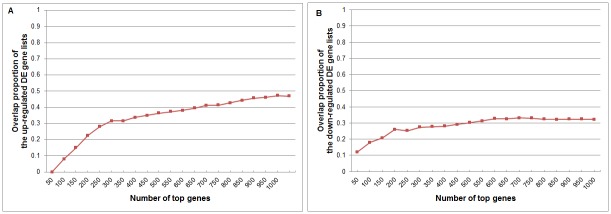
Overlap proportions of differentially expressed genes detected by SAM from the data with corrected and uncorrected global shift effects on Loven et al’s data. (A) Up-regulated DE genes. (B) Down-regulated DE genes. The settings are the same with Fig 2.

Fig 3 shows the results of SAM on the same data. We can see that the overlap is much poorer and more complicated. There is almost no overlap among the top genes. This tells that the global shift causes more changes in the variances of gene expression as the mean shifted, especially when sample size is very small.

### Results on Simulated Datasets

**Fig 4:**
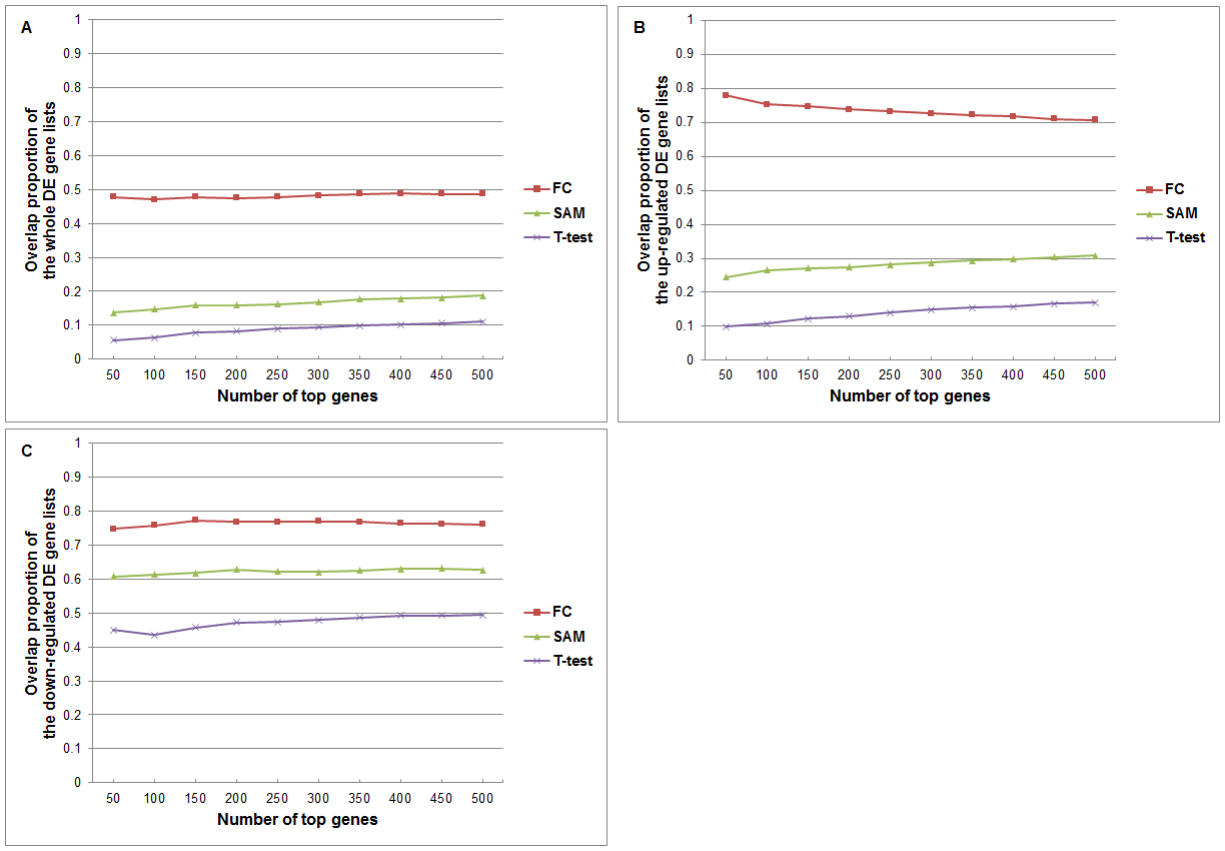
Overlap proportions of differentially expressed genes detected by fold-change, SAM and t-test from the data with simulated global shift effects, averaged over the 20 datasets. (A) DE genes ranked by whole differentially expressed differences; (B) Up-regulated DE genes; (C) Down-regulated DE genes. The settings are the same with Fig 2.

For each of the 20 simulated datasets, we applied the same analysis on the sister datasets and compared the overlap between the top 50, 100, …, 500 genes in the whole differentially expressed gene lists, and also in the separated lists of up-regulated DE genes and down-regulated DE genes. All the results are provided in the S1 File. We averaged overlap proportions on the 20 datasets for the fold-change, t-test and SAM methods. Fig 4 shows the results.

We can see that for the top genes selected by fold-change, the overlap proportion can be kept at the level of 70–90% for both the up-regulated gene list and down-regulated gene list. However, for statistical-test methods like t-test and SAM, overlaps between results on sister datasets are very low. The overlap proportions obtained by SAM are slightly higher than that by t-test. We also see that the overlaps in the down-regulated genes are relatively higher in the case of global amplification effect in the disease group. We can imagine that the overlaps in the up-regulated genes will be relatively higher when the global shift is de-amplification in the disease group.

From the result of the above experiments, we found that methods for detecting differential gene expression based on fold-change are more robust to global expression shift than methods based on statistical tests, and the overlap proportion of FC is rather high. We can use the ideal example of Table 1 to illustrate the reason behind the above observations.

Table 3 shows the fold-change (FC) ratios in the two experiments in the toy example of Table 1, plus an extra example (Experiment #3) for the situation of de-amplification (shift factor = 0.5). In Experiment #1 with global shift effect factor 2, the FC of a gene that has equal expression in each cell of the test group and the control group will be 0.5. A real down-regulated gene will have FC less than 0.5, and a real up-regulated gene will have FC larger than 0.5. Therefore, genes with observed FC between 0.5 to 1.0 will be mistaken as down-regulated in the detection. In Experiment #2 that does not suffer from global shift as the numbers of cells in the two groups have been controlled to be equal, a gene with no differential expression will give the FC of 1.0, and a down-regulated or up-regulated gene will give FC smaller than 1 or larger than 1, respectively. For experiment #3 with global shift effect factor 0.5, the FC of a gene that has equal expression in each cell of the test group and the control group will be 2. A real down-regulated gene will have FC less than 2, and a real up-regulated gene will have FC larger than 2. This will cause genes with detected FC between 1.0 to 2.0 be mistaken as up-regulated in the detection. For genes with very high or very low FC on top of the DE gene lists, they tend to be detected correctly regardless to the global shift effect.

**Table 3:** Illustrative examples of experiments without global shift and with shifts of two directions.

Experiment #1: shift factor 2.0			
FC value	*< 0*.*5*	0.5~1	*>1*
Identification / Truth	*down / down*	down / up	*up / up*
Experiment #2: no global shift			
FC value	*< 1*	-	*>1*
Identification / Truth	*down / down*	-	*up / up*
Experiment #3: shift factor 0.5			
FC value	*<1*	1~2	*>2*
Identification / Truth	*down / down*	up / down	*up / up*

FC, fold-change ratio; down, down-regulated gene; up, up-regulated gene.

From this example, we can draw some conclusions in some specific circumstances for deterministic methods based on the relative rank of genes in their expression, such as fold-change method. We can see that in situations highlighted in *italic* in Table 3, a gene’s up- or down-regulation can be identified correctly by fold-change. Although global-shift may change the specific value of fold-change-ratio, the direction of the change is not affected for those situations. This explains why the top genes in the DE gene lists by fold-change are not much affected by the global shift in our experiments.

When we consider variations among multiple samples in each group, statistical tests need to be adopted to infer whether a gene has significant differences in its expression between the two groups. The differences in means of the expression between the two groups need to be normalized by the variance. Tables 4 and 5 show illustrative examples of the expression of 4 toy genes in two groups of samples in situations of no global shift and with global shift factor of 2.0. The t-test results for each gene in the two situations are provided. We can see that when there is global shift in one of the two groups, both the difference of means and the pooled variance have dramatic changes. This results in big changes in the inference and the rank of the genes by their p-values. The influence of global shift on SAM will also be similar. The *s*_*0*_ parameter in SAM makes it less sensitive to changes in the estimated variance, so SAM is slightly more robust to global shift effect as we seen in the experiments.

**Table 4:** Illustrative examples of multiple samples of the experiment with no shift effect.

Gene	Expression in control samples	Expression in test samples	Difference of means	Pooled variance	t	p	Inference (p-value<0.05)	p-value rank (from small to large)
#1	150, 200, 250	1, 50, 100	149.67	40.62	3.68	0.021	Significant, Down	3
#2	101.1, 101.2, 101.3	100.1, 100.2, 100.3	1	0.082	12.25	2.6e-04	Significant, Down	1
#3	150, 200, 250	50, 100, 150	100	40.82	2.45	0.07	Non-significant	4
#4	180, 200, 220	95.1, 100.2, 105.3	99.8	11.92	8.37	0.0096	Significant, Down	2

**Table 5:** Illustrative examples of multiple samples of the experiment with shift factor = 2 in test samples.

Gene	Expression in control samples	Expression in test samples	Difference of means	Pooled variance	t	p	Inference (p-value<0.05)	p-value rank (from small to large)
#1	150, 200, 250	2, 100, 200	99.33	64.03	1.55	0.220	Non-significant	2
#2	101.1, 101.2, 101.3	200.2, 200.4, 200.6	-99.2	0.13	-768.4	6.81e-09	Significant, Up	1
#3	150, 200, 250	100, 200, 300	0	64.55	0	1	Non-significant	4
#4	180, 200, 220	190.2, 200.4, 210.6	-0.4	12.96	-0.03	0.97	Non-significant	3

### Results on Sample Classification and Gene Selection

We applied R-SVM and SVM-RFE on the sister data of the 20 datasets for the classification of the two groups and selection of informative genes for the classification. The results of two methods are similar, so we only present the results of R-SVM here. We compared the leave-one-out cross-validation errors on the sister datasets and the overlap between the two gene lists selected on the sister datasets. Tables 6 and 7 give the error rates at different gene-selection levels on Dataset 1 and Dataset 2. Fig 5 shows the overlap of selected genes in the sister datasets at different gene-selection levels. Results on the other datasets are provided in the S2 File. Without surprise, we can see the classification error becomes smaller (0 for most of the data in our experiments) when there is global shift in one of the two groups, since the global shift brings systematic difference in gene expression between the two groups and makes them more separable. However, the overlap between the genes selected from sister datasets is low, especially when we select only a small number of genes.

**Table 6:** The classification errors of rank lists of Dataset 1.

# of genes	21649	10824	5412	2706	1353	676	338	169	84	42	21	10	5
On original data	0.047	0.07	0.047	0.047	0.047	0.07	0.07	0.07	0.07	0.07	0.07	0.047	0.047
On data with shift factor 2	0	0	0	0	0	0	0	0	0	0	0	0	0

**Table 7:** The classification errors of rank lists of Dataset 2.

# of genes	54429	27214	13607	6804	3402	1701	850	425	212	106	53	26	13	6
On original data	0.115	0.098	0.098	0.098	0.098	0.098	0.098	0.115	0.082	0.098	0.082	0.082	0.082	0.082
On data with shift factor 2	0	0	0	0	0	0	0	0	0	0	0	0	0	0

**Fig 5:**
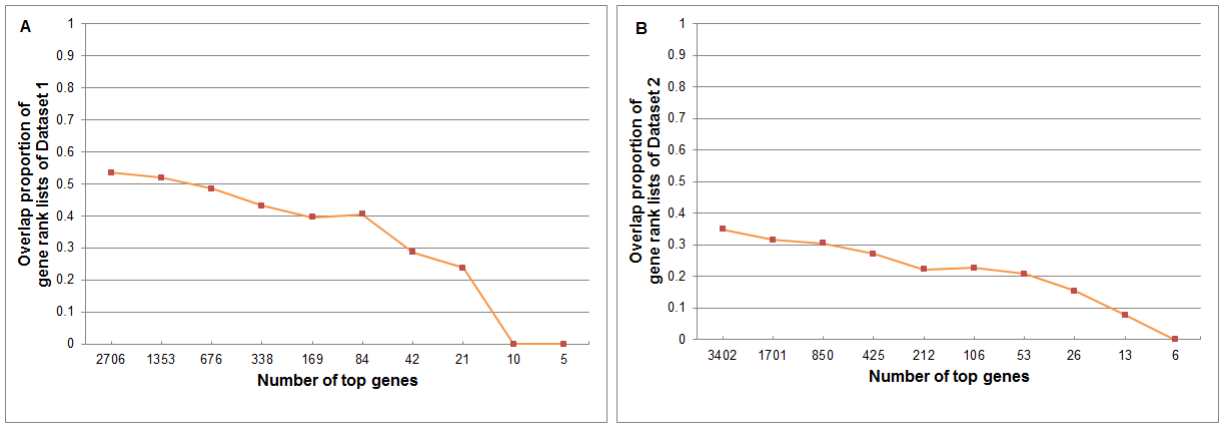
The overlap proportion of selected gene lists by R-SVM. (A) on Dataset 1; (B) on Dataset 2. The settings are the same with Fig 2.

## Conclusion

We studied the influence of the global gene expression shift on downstream analyses of differentially expressed genes, sample classification and gene selection. We designed a set of experiments to study the influence of the shift effect on downstream analyses based on data with known global shift effect and also generated data to simulate different situations of the effect. The experiments as well as study on illustrative toy-data models show that deterministic methods for detecting differential expression such as fold-change are less sensitive to global shift in gene expression, although they have the obvious shortcoming for not being able to provide information on the significance of differential expression. The top genes selected according to their rank of fold-change are quite robust with regard to global shift. On the other hand, statistical methods like t-test and SAM suffer severely from global shift effects. The majority of the top genes can be changed if global shift effect exists in the data and cannot be corrected. For sample classification and gene selection with machine learning approaches, the classification accuracy is increased by the global shift, but the genes selected with the classification can be severely affected by global shift.

The DE gene detect methods we used were among the simplest methods that have been widely used by most published work. They are the basis for more sophisticated methods. Therefore observations on these simple methods also shed light on more sophisticated methods, and also on classification methods based on filtering-type of feature selection. From the observations of this study, we can see that great caution should be taken in analyzing gene expression data when there can be abnormal expression of master regulator genes like c-MYC that may cause global expression shift. As pointed out in Loven et al’s work, spike-in signals that can provide control on the number of cells will be the optimal solution to avoid possible biased conclusions introduced by uncorrected global shift effect. When such signals are unavailable, methods need to be developed to test the existence of global shift and estimate the shift factor so that the influence can be corrected. Biological knowledge about the expression and function of master regulators in multiple tissues will also be helpful for estimating the global shift effect.

## Supporting Information

S1 File Results of overlap proportions of DE genes on 20 datasets. (DOCX)

S2 File Results of sample classification and gene selection on the 20 datasets. (XLSX)
