# Supplementary material for: The Influence of the Global Gene Expression Shift on Downstream Analyses
Source: PLoS One. 2016 Apr 19;11(4):e0153903. doi: 10.1371/journal.pone.0153903 (PMC4836657; doi:10.1371/journal.pone.0153903)
Supplement: S1 File — (DOCX) [file pone.0153903.s001.docx]

S1 File - Results of overlap proportions of DE genes on 20 datasets

Table 1. Overlap proportions of whole differentially expressed genes of t-test.

| Data ID | 50 | 100 | 150 | 200 | 250 | 300 | 350 | 400 | 450 | 500 |
| --- | --- | --- | --- | --- | --- | --- | --- | --- | --- | --- |
| 1 | 0.060 | 0.130 | 0.153 | 0.170 | 0.184 | 0.190 | 0.206 | 0.195 | 0.204 | 0.206 |
| 2 | 0.040 | 0.030 | 0.033 | 0.040 | 0.032 | 0.033 | 0.034 | 0.050 | 0.058 | 0.058 |
| 3 | 0.220 | 0.230 | 0.280 | 0.320 | 0.312 | 0.313 | 0.334 | 0.333 | 0.331 | 0.330 |
| 4 | 0.220 | 0.220 | 0.253 | 0.235 | 0.260 | 0.257 | 0.243 | 0.260 | 0.264 | 0.272 |
| 5 | 0.220 | 0.240 | 0.247 | 0.250 | 0.256 | 0.253 | 0.251 | 0.240 | 0.238 | 0.242 |
| 6 | 0.140 | 0.110 | 0.167 | 0.160 | 0.172 | 0.207 | 0.206 | 0.208 | 0.213 | 0.222 |
| 7 | 0.000 | 0.020 | 0.033 | 0.035 | 0.076 | 0.090 | 0.103 | 0.105 | 0.120 | 0.132 |
| 8 | 0.000 | 0.000 | 0.000 | 0.000 | 0.000 | 0.000 | 0.003 | 0.005 | 0.007 | 0.006 |
| 9 | 0.000 | 0.000 | 0.000 | 0.000 | 0.000 | 0.000 | 0.000 | 0.000 | 0.000 | 0.000 |
| 10 | 0.020 | 0.010 | 0.007 | 0.005 | 0.004 | 0.007 | 0.006 | 0.008 | 0.009 | 0.014 |
| 11 | 0.000 | 0.000 | 0.007 | 0.010 | 0.008 | 0.007 | 0.006 | 0.005 | 0.007 | 0.006 |
| 12 | 0.000 | 0.010 | 0.033 | 0.030 | 0.044 | 0.053 | 0.063 | 0.070 | 0.078 | 0.082 |
| 13 | 0.000 | 0.000 | 0.000 | 0.000 | 0.000 | 0.000 | 0.000 | 0.000 | 0.000 | 0.002 |
| 14 | 0.000 | 0.010 | 0.007 | 0.005 | 0.004 | 0.003 | 0.003 | 0.003 | 0.002 | 0.004 |
| 15 | 0.040 | 0.050 | 0.060 | 0.060 | 0.072 | 0.080 | 0.094 | 0.113 | 0.122 | 0.126 |
| 16 | 0.000 | 0.040 | 0.060 | 0.075 | 0.096 | 0.103 | 0.100 | 0.108 | 0.120 | 0.132 |
| 17 | 0.000 | 0.000 | 0.000 | 0.000 | 0.012 | 0.017 | 0.017 | 0.018 | 0.018 | 0.018 |
| 18 | 0.020 | 0.020 | 0.040 | 0.060 | 0.060 | 0.060 | 0.069 | 0.075 | 0.084 | 0.088 |
| 19 | 0.140 | 0.140 | 0.140 | 0.135 | 0.132 | 0.143 | 0.143 | 0.155 | 0.169 | 0.178 |
| 20 | 0.000 | 0.020 | 0.047 | 0.055 | 0.068 | 0.080 | 0.080 | 0.085 | 0.087 | 0.096 |

Table 2. Overlap proportions of up-regulated DE genes of t-test.

| Data ID | 50 | 100 | 150 | 200 | 250 | 300 | 350 | 400 | 450 | 500 |
| --- | --- | --- | --- | --- | --- | --- | --- | --- | --- | --- |
| 1 | 0.080 | 0.150 | 0.193 | 0.195 | 0.220 | 0.223 | 0.226 | 0.218 | 0.227 | 0.240 |
| 2 | 0.040 | 0.030 | 0.047 | 0.045 | 0.040 | 0.040 | 0.046 | 0.063 | 0.078 | 0.086 |
| 3 | 0.220 | 0.320 | 0.373 | 0.390 | 0.392 | 0.403 | 0.420 | 0.430 | 0.447 | 0.444 |
| 4 | 0.260 | 0.260 | 0.267 | 0.280 | 0.296 | 0.293 | 0.294 | 0.313 | 0.313 | 0.314 |
| 5 | 0.360 | 0.360 | 0.380 | 0.340 | 0.368 | 0.367 | 0.349 | 0.330 | 0.340 | 0.340 |
| 6 | 0.500 | 0.450 | 0.453 | 0.505 | 0.528 | 0.547 | 0.546 | 0.540 | 0.536 | 0.532 |
| 7 | 0.120 | 0.100 | 0.120 | 0.130 | 0.152 | 0.187 | 0.191 | 0.190 | 0.202 | 0.212 |
| 8 | 0.000 | 0.000 | 0.000 | 0.000 | 0.000 | 0.000 | 0.006 | 0.005 | 0.007 | 0.006 |
| 9 | 0.000 | 0.000 | 0.000 | 0.000 | 0.000 | 0.000 | 0.000 | 0.000 | 0.000 | 0.000 |
| 10 | 0.020 | 0.010 | 0.007 | 0.005 | 0.004 | 0.007 | 0.006 | 0.013 | 0.013 | 0.016 |
| 11 | 0.000 | 0.000 | 0.007 | 0.010 | 0.008 | 0.007 | 0.006 | 0.005 | 0.007 | 0.006 |
| 12 | 0.000 | 0.040 | 0.067 | 0.060 | 0.080 | 0.097 | 0.120 | 0.128 | 0.147 | 0.162 |
| 13 | 0.000 | 0.000 | 0.000 | 0.000 | 0.000 | 0.000 | 0.003 | 0.005 | 0.013 | 0.012 |
| 14 | 0.000 | 0.010 | 0.007 | 0.005 | 0.004 | 0.003 | 0.003 | 0.005 | 0.004 | 0.004 |
| 15 | 0.080 | 0.070 | 0.080 | 0.105 | 0.124 | 0.163 | 0.177 | 0.200 | 0.213 | 0.228 |
| 16 | 0.100 | 0.140 | 0.200 | 0.220 | 0.272 | 0.290 | 0.320 | 0.318 | 0.329 | 0.338 |
| 17 | 0.000 | 0.000 | 0.000 | 0.000 | 0.012 | 0.017 | 0.017 | 0.018 | 0.018 | 0.018 |
| 18 | 0.020 | 0.020 | 0.040 | 0.060 | 0.060 | 0.060 | 0.071 | 0.080 | 0.096 | 0.090 |
| 19 | 0.160 | 0.160 | 0.147 | 0.160 | 0.172 | 0.197 | 0.197 | 0.210 | 0.224 | 0.244 |
| 20 | 0.000 | 0.030 | 0.053 | 0.075 | 0.072 | 0.080 | 0.086 | 0.098 | 0.104 | 0.106 |

Table 3. Overlap proportions of down-regulated DE genes of t-test.

| Data ID | 50 | 100 | 150 | 200 | 250 | 300 | 350 | 400 | 450 | 500 |
| --- | --- | --- | --- | --- | --- | --- | --- | --- | --- | --- |
| 1 | 0.340 | 0.350 | 0.393 | 0.385 | 0.376 | 0.373 | 0.377 | 0.370 | 0.367 | 0.360 |
| 2 | 0.280 | 0.210 | 0.220 | 0.265 | 0.296 | 0.307 | 0.317 | 0.328 | 0.327 | 0.348 |
| 3 | 0.820 | 0.640 | 0.680 | 0.670 | 0.624 | 0.623 | 0.646 | 0.635 | 0.629 | 0.618 |
| 4 | 0.640 | 0.550 | 0.560 | 0.565 | 0.532 | 0.523 | 0.497 | 0.513 | 0.511 | 0.506 |
| 5 | 0.700 | 0.750 | 0.693 | 0.680 | 0.656 | 0.660 | 0.646 | 0.633 | 0.627 | 0.620 |
| 6 | 0.740 | 0.670 | 0.760 | 0.705 | 0.724 | 0.737 | 0.740 | 0.738 | 0.733 | 0.726 |
| 7 | 0.660 | 0.560 | 0.553 | 0.605 | 0.648 | 0.640 | 0.657 | 0.650 | 0.653 | 0.664 |
| 8 | 0.400 | 0.440 | 0.487 | 0.505 | 0.528 | 0.527 | 0.546 | 0.550 | 0.551 | 0.554 |
| 9 | 0.420 | 0.500 | 0.520 | 0.560 | 0.544 | 0.537 | 0.554 | 0.573 | 0.582 | 0.602 |
| 10 | 0.600 | 0.570 | 0.553 | 0.560 | 0.552 | 0.567 | 0.586 | 0.595 | 0.593 | 0.592 |
| 11 | 0.560 | 0.530 | 0.520 | 0.510 | 0.472 | 0.483 | 0.480 | 0.490 | 0.489 | 0.482 |
| 12 | 0.260 | 0.290 | 0.367 | 0.410 | 0.444 | 0.470 | 0.483 | 0.513 | 0.502 | 0.506 |
| 13 | 0.540 | 0.510 | 0.627 | 0.670 | 0.696 | 0.693 | 0.703 | 0.703 | 0.704 | 0.686 |
| 14 | 0.120 | 0.180 | 0.180 | 0.200 | 0.212 | 0.227 | 0.214 | 0.210 | 0.216 | 0.212 |
| 15 | 0.580 | 0.620 | 0.627 | 0.650 | 0.648 | 0.657 | 0.651 | 0.678 | 0.658 | 0.666 |
| 16 | 0.720 | 0.620 | 0.653 | 0.675 | 0.696 | 0.700 | 0.723 | 0.713 | 0.716 | 0.728 |
| 17 | 0.040 | 0.070 | 0.087 | 0.140 | 0.136 | 0.133 | 0.154 | 0.153 | 0.167 | 0.174 |
| 18 | 0.320 | 0.340 | 0.353 | 0.355 | 0.328 | 0.360 | 0.383 | 0.413 | 0.418 | 0.412 |
| 19 | 0.200 | 0.230 | 0.247 | 0.235 | 0.256 | 0.257 | 0.269 | 0.270 | 0.276 | 0.280 |
| 20 | 0.060 | 0.060 | 0.073 | 0.080 | 0.084 | 0.097 | 0.117 | 0.128 | 0.133 | 0.146 |

Table 4. Overlap proportions of whole differentially expressed genes of SAM.

| Data ID | 50 | 100 | 150 | 200 | 250 | 300 | 350 | 400 | 450 | 500 |
| --- | --- | --- | --- | --- | --- | --- | --- | --- | --- | --- |
| 1 | 0.060 | 0.180 | 0.200 | 0.225 | 0.240 | 0.260 | 0.277 | 0.293 | 0.296 | 0.308 |
| 2 | 0.120 | 0.180 | 0.193 | 0.180 | 0.196 | 0.197 | 0.197 | 0.203 | 0.209 | 0.214 |
| 3 | 0.360 | 0.390 | 0.427 | 0.405 | 0.376 | 0.383 | 0.409 | 0.408 | 0.409 | 0.424 |
| 4 | 0.340 | 0.360 | 0.373 | 0.380 | 0.348 | 0.353 | 0.351 | 0.340 | 0.340 | 0.346 |
| 5 | 0.480 | 0.420 | 0.393 | 0.410 | 0.396 | 0.387 | 0.397 | 0.385 | 0.378 | 0.370 |
| 6 | 0.440 | 0.320 | 0.347 | 0.360 | 0.360 | 0.370 | 0.377 | 0.380 | 0.384 | 0.376 |
| 7 | 0.000 | 0.040 | 0.060 | 0.080 | 0.084 | 0.113 | 0.114 | 0.115 | 0.116 | 0.116 |
| 8 | 0.000 | 0.010 | 0.007 | 0.005 | 0.008 | 0.020 | 0.029 | 0.028 | 0.040 | 0.040 |
| 9 | 0.000 | 0.000 | 0.000 | 0.000 | 0.000 | 0.000 | 0.000 | 0.005 | 0.004 | 0.010 |
| 10 | 0.000 | 0.020 | 0.027 | 0.030 | 0.056 | 0.050 | 0.046 | 0.048 | 0.049 | 0.046 |
| 11 | 0.020 | 0.010 | 0.007 | 0.005 | 0.008 | 0.010 | 0.014 | 0.013 | 0.013 | 0.012 |
| 12 | 0.080 | 0.060 | 0.093 | 0.080 | 0.088 | 0.103 | 0.106 | 0.108 | 0.109 | 0.118 |
| 13 | 0.000 | 0.000 | 0.000 | 0.000 | 0.000 | 0.000 | 0.000 | 0.003 | 0.004 | 0.010 |
| 14 | 0.000 | 0.000 | 0.000 | 0.000 | 0.004 | 0.007 | 0.009 | 0.018 | 0.024 | 0.036 |
| 15 | 0.080 | 0.100 | 0.127 | 0.125 | 0.128 | 0.137 | 0.151 | 0.160 | 0.169 | 0.180 |
| 16 | 0.120 | 0.190 | 0.200 | 0.205 | 0.220 | 0.257 | 0.274 | 0.283 | 0.273 | 0.290 |
| 17 | 0.000 | 0.000 | 0.000 | 0.000 | 0.000 | 0.000 | 0.000 | 0.005 | 0.007 | 0.008 |
| 18 | 0.040 | 0.080 | 0.073 | 0.060 | 0.056 | 0.070 | 0.083 | 0.090 | 0.091 | 0.106 |
| 19 | 0.420 | 0.410 | 0.467 | 0.450 | 0.464 | 0.430 | 0.460 | 0.458 | 0.462 | 0.470 |
| 20 | 0.180 | 0.190 | 0.180 | 0.190 | 0.204 | 0.220 | 0.231 | 0.243 | 0.262 | 0.276 |

Table 5. Overlap proportions of up-regulated DE genes of SAM.

| Data ID | 50 | 100 | 150 | 200 | 250 | 300 | 350 | 400 | 450 | 500 |
| --- | --- | --- | --- | --- | --- | --- | --- | --- | --- | --- |
| 1 | 0.160 | 0.220 | 0.247 | 0.295 | 0.304 | 0.333 | 0.331 | 0.345 | 0.356 | 0.362 |
| 2 | 0.120 | 0.200 | 0.213 | 0.220 | 0.232 | 0.233 | 0.240 | 0.243 | 0.262 | 0.282 |
| 3 | 0.560 | 0.530 | 0.520 | 0.505 | 0.508 | 0.527 | 0.560 | 0.553 | 0.551 | 0.554 |
| 4 | 0.520 | 0.470 | 0.467 | 0.475 | 0.456 | 0.457 | 0.466 | 0.453 | 0.451 | 0.454 |
| 5 | 0.880 | 0.890 | 0.893 | 0.850 | 0.876 | 0.867 | 0.849 | 0.850 | 0.853 | 0.852 |
| 6 | 0.780 | 0.870 | 0.840 | 0.840 | 0.840 | 0.827 | 0.823 | 0.835 | 0.838 | 0.844 |
| 7 | 0.080 | 0.130 | 0.133 | 0.215 | 0.232 | 0.260 | 0.266 | 0.278 | 0.278 | 0.272 |
| 8 | 0.000 | 0.010 | 0.007 | 0.005 | 0.016 | 0.033 | 0.037 | 0.038 | 0.058 | 0.056 |
| 9 | 0.000 | 0.000 | 0.000 | 0.000 | 0.004 | 0.003 | 0.003 | 0.005 | 0.007 | 0.012 |
| 10 | 0.020 | 0.020 | 0.027 | 0.040 | 0.060 | 0.053 | 0.046 | 0.050 | 0.053 | 0.052 |
| 11 | 0.020 | 0.010 | 0.007 | 0.010 | 0.012 | 0.010 | 0.014 | 0.013 | 0.013 | 0.012 |
| 12 | 0.100 | 0.130 | 0.167 | 0.160 | 0.188 | 0.203 | 0.211 | 0.235 | 0.242 | 0.250 |
| 13 | 0.000 | 0.000 | 0.000 | 0.005 | 0.016 | 0.027 | 0.034 | 0.043 | 0.053 | 0.060 |
| 14 | 0.000 | 0.000 | 0.020 | 0.025 | 0.032 | 0.027 | 0.034 | 0.043 | 0.049 | 0.054 |
| 15 | 0.300 | 0.330 | 0.353 | 0.345 | 0.356 | 0.367 | 0.394 | 0.400 | 0.398 | 0.406 |
| 16 | 0.600 | 0.710 | 0.700 | 0.670 | 0.644 | 0.617 | 0.637 | 0.633 | 0.627 | 0.630 |
| 17 | 0.000 | 0.000 | 0.000 | 0.000 | 0.000 | 0.000 | 0.000 | 0.005 | 0.007 | 0.008 |
| 18 | 0.040 | 0.080 | 0.073 | 0.060 | 0.056 | 0.077 | 0.086 | 0.095 | 0.107 | 0.120 |
| 19 | 0.520 | 0.530 | 0.573 | 0.570 | 0.608 | 0.603 | 0.609 | 0.590 | 0.596 | 0.590 |
| 20 | 0.180 | 0.190 | 0.180 | 0.190 | 0.204 | 0.220 | 0.234 | 0.245 | 0.271 | 0.278 |

Table 6. Overlap proportions of down-regulated DE genes of SAM.

| Data ID | 50 | 100 | 150 | 200 | 250 | 300 | 350 | 400 | 450 | 500 |
| --- | --- | --- | --- | --- | --- | --- | --- | --- | --- | --- |
| 1 | 0.780 | 0.740 | 0.687 | 0.650 | 0.652 | 0.630 | 0.626 | 0.635 | 0.616 | 0.608 |
| 2 | 0.460 | 0.380 | 0.413 | 0.420 | 0.448 | 0.470 | 0.486 | 0.503 | 0.516 | 0.528 |
| 3 | 0.860 | 0.780 | 0.813 | 0.815 | 0.752 | 0.757 | 0.749 | 0.735 | 0.738 | 0.714 |
| 4 | 0.840 | 0.770 | 0.733 | 0.745 | 0.724 | 0.713 | 0.700 | 0.688 | 0.691 | 0.670 |
| 5 | 0.900 | 0.870 | 0.873 | 0.910 | 0.908 | 0.887 | 0.891 | 0.868 | 0.880 | 0.882 |
| 6 | 0.840 | 0.750 | 0.740 | 0.765 | 0.792 | 0.763 | 0.760 | 0.788 | 0.784 | 0.792 |
| 7 | 0.860 | 0.850 | 0.847 | 0.805 | 0.816 | 0.817 | 0.814 | 0.810 | 0.791 | 0.804 |
| 8 | 0.580 | 0.700 | 0.707 | 0.690 | 0.696 | 0.677 | 0.674 | 0.668 | 0.682 | 0.666 |
| 9 | 0.640 | 0.670 | 0.647 | 0.670 | 0.636 | 0.647 | 0.649 | 0.675 | 0.667 | 0.680 |
| 10 | 0.640 | 0.630 | 0.627 | 0.650 | 0.640 | 0.660 | 0.669 | 0.675 | 0.676 | 0.664 |
| 11 | 0.680 | 0.680 | 0.667 | 0.660 | 0.640 | 0.607 | 0.600 | 0.610 | 0.613 | 0.608 |
| 12 | 0.340 | 0.380 | 0.413 | 0.415 | 0.452 | 0.473 | 0.483 | 0.510 | 0.522 | 0.520 |
| 13 | 0.700 | 0.700 | 0.753 | 0.805 | 0.760 | 0.757 | 0.771 | 0.775 | 0.773 | 0.774 |
| 14 | 0.220 | 0.350 | 0.340 | 0.345 | 0.348 | 0.343 | 0.351 | 0.355 | 0.353 | 0.358 |
| 15 | 0.720 | 0.820 | 0.773 | 0.780 | 0.796 | 0.777 | 0.774 | 0.778 | 0.782 | 0.764 |
| 16 | 0.800 | 0.770 | 0.767 | 0.790 | 0.768 | 0.797 | 0.820 | 0.813 | 0.813 | 0.812 |
| 17 | 0.300 | 0.440 | 0.447 | 0.450 | 0.412 | 0.387 | 0.400 | 0.400 | 0.389 | 0.382 |
| 18 | 0.440 | 0.450 | 0.513 | 0.590 | 0.608 | 0.640 | 0.634 | 0.638 | 0.651 | 0.640 |
| 19 | 0.400 | 0.390 | 0.413 | 0.415 | 0.412 | 0.390 | 0.423 | 0.410 | 0.400 | 0.396 |
| 20 | 0.160 | 0.160 | 0.193 | 0.190 | 0.188 | 0.213 | 0.229 | 0.255 | 0.278 | 0.284 |

Table 7. Overlap proportions of whole differentially expressed genes of fold-change ratio.

| Data ID | 50 | 100 | 150 | 200 | 250 | 300 | 350 | 400 | 450 | 500 |
| --- | --- | --- | --- | --- | --- | --- | --- | --- | --- | --- |
| 1 | 0.420 | 0.430 | 0.473 | 0.500 | 0.492 | 0.513 | 0.529 | 0.545 | 0.547 | 0.554 |
| 2 | 0.660 | 0.620 | 0.580 | 0.580 | 0.584 | 0.577 | 0.563 | 0.545 | 0.527 | 0.526 |
| 3 | 0.520 | 0.580 | 0.533 | 0.530 | 0.540 | 0.547 | 0.560 | 0.563 | 0.573 | 0.588 |
| 4 | 0.400 | 0.520 | 0.513 | 0.545 | 0.560 | 0.580 | 0.577 | 0.578 | 0.580 | 0.582 |
| 5 | 0.500 | 0.420 | 0.467 | 0.470 | 0.488 | 0.517 | 0.477 | 0.473 | 0.464 | 0.444 |
| 6 | 0.440 | 0.490 | 0.520 | 0.495 | 0.476 | 0.497 | 0.497 | 0.485 | 0.480 | 0.484 |
| 7 | 0.380 | 0.420 | 0.413 | 0.405 | 0.412 | 0.417 | 0.446 | 0.463 | 0.484 | 0.492 |
| 8 | 0.400 | 0.370 | 0.393 | 0.410 | 0.448 | 0.443 | 0.451 | 0.458 | 0.469 | 0.468 |
| 9 | 0.300 | 0.340 | 0.320 | 0.315 | 0.340 | 0.353 | 0.360 | 0.360 | 0.384 | 0.384 |
| 10 | 0.460 | 0.400 | 0.407 | 0.375 | 0.376 | 0.370 | 0.383 | 0.395 | 0.396 | 0.404 |
| 11 | 0.580 | 0.590 | 0.573 | 0.575 | 0.604 | 0.603 | 0.606 | 0.618 | 0.611 | 0.596 |
| 12 | 0.260 | 0.240 | 0.280 | 0.285 | 0.284 | 0.287 | 0.283 | 0.283 | 0.273 | 0.278 |
| 13 | 0.260 | 0.290 | 0.340 | 0.325 | 0.328 | 0.353 | 0.369 | 0.373 | 0.367 | 0.370 |
| 14 | 0.160 | 0.130 | 0.113 | 0.140 | 0.148 | 0.157 | 0.171 | 0.170 | 0.160 | 0.152 |
| 15 | 0.520 | 0.470 | 0.473 | 0.455 | 0.444 | 0.430 | 0.449 | 0.440 | 0.447 | 0.446 |
| 16 | 0.440 | 0.450 | 0.493 | 0.490 | 0.484 | 0.473 | 0.483 | 0.483 | 0.464 | 0.472 |
| 17 | 0.800 | 0.770 | 0.820 | 0.790 | 0.792 | 0.780 | 0.797 | 0.790 | 0.771 | 0.778 |
| 18 | 0.840 | 0.820 | 0.767 | 0.770 | 0.724 | 0.737 | 0.734 | 0.740 | 0.736 | 0.724 |
| 19 | 0.460 | 0.370 | 0.333 | 0.330 | 0.332 | 0.343 | 0.329 | 0.320 | 0.316 | 0.320 |
| 20 | 0.760 | 0.700 | 0.747 | 0.705 | 0.684 | 0.660 | 0.666 | 0.683 | 0.678 | 0.680 |

Table 8. Overlap proportions of up-regulated DE genes of fold-change ratio.

| Data ID | 50 | 100 | 150 | 200 | 250 | 300 | 350 | 400 | 450 | 500 |
| --- | --- | --- | --- | --- | --- | --- | --- | --- | --- | --- |
| 1 | 0.720 | 0.650 | 0.640 | 0.660 | 0.652 | 0.640 | 0.643 | 0.648 | 0.647 | 0.648 |
| 2 | 0.880 | 0.860 | 0.820 | 0.840 | 0.876 | 0.850 | 0.834 | 0.820 | 0.796 | 0.796 |
| 3 | 0.840 | 0.850 | 0.840 | 0.850 | 0.836 | 0.817 | 0.837 | 0.823 | 0.813 | 0.812 |
| 4 | 0.840 | 0.820 | 0.833 | 0.815 | 0.784 | 0.770 | 0.783 | 0.790 | 0.798 | 0.796 |
| 5 | 0.900 | 0.930 | 0.907 | 0.900 | 0.876 | 0.850 | 0.886 | 0.873 | 0.847 | 0.838 |
| 6 | 0.940 | 0.910 | 0.887 | 0.880 | 0.888 | 0.883 | 0.871 | 0.863 | 0.858 | 0.866 |
| 7 | 0.900 | 0.810 | 0.820 | 0.800 | 0.828 | 0.797 | 0.769 | 0.765 | 0.771 | 0.786 |
| 8 | 0.700 | 0.690 | 0.747 | 0.755 | 0.736 | 0.717 | 0.703 | 0.700 | 0.696 | 0.688 |
| 9 | 0.660 | 0.650 | 0.627 | 0.605 | 0.604 | 0.617 | 0.609 | 0.600 | 0.593 | 0.580 |
| 10 | 0.820 | 0.670 | 0.620 | 0.565 | 0.580 | 0.570 | 0.554 | 0.555 | 0.556 | 0.550 |
| 11 | 0.840 | 0.730 | 0.773 | 0.770 | 0.764 | 0.767 | 0.751 | 0.748 | 0.744 | 0.740 |
| 12 | 0.740 | 0.700 | 0.673 | 0.630 | 0.624 | 0.590 | 0.571 | 0.560 | 0.558 | 0.572 |
| 13 | 0.800 | 0.760 | 0.720 | 0.685 | 0.700 | 0.713 | 0.686 | 0.693 | 0.691 | 0.680 |
| 14 | 0.200 | 0.170 | 0.167 | 0.195 | 0.192 | 0.190 | 0.191 | 0.188 | 0.180 | 0.172 |
| 15 | 0.860 | 0.880 | 0.827 | 0.840 | 0.784 | 0.783 | 0.794 | 0.785 | 0.760 | 0.748 |
| 16 | 0.820 | 0.880 | 0.873 | 0.870 | 0.844 | 0.863 | 0.849 | 0.820 | 0.809 | 0.812 |
| 17 | 0.840 | 0.840 | 0.880 | 0.870 | 0.872 | 0.863 | 0.869 | 0.858 | 0.844 | 0.838 |
| 18 | 0.840 | 0.920 | 0.867 | 0.860 | 0.868 | 0.883 | 0.883 | 0.878 | 0.880 | 0.870 |
| 19 | 0.680 | 0.610 | 0.647 | 0.640 | 0.644 | 0.670 | 0.680 | 0.685 | 0.680 | 0.664 |
| 20 | 0.780 | 0.740 | 0.773 | 0.740 | 0.704 | 0.693 | 0.694 | 0.710 | 0.707 | 0.708 |

Table 9. Overlap proportions of down-regulated DE genes of fold-change ratio.

| Data ID | 50 | 100 | 150 | 200 | 250 | 300 | 350 | 400 | 450 | 500 |
| --- | --- | --- | --- | --- | --- | --- | --- | --- | --- | --- |
| 1 | 0.760 | 0.780 | 0.773 | 0.765 | 0.736 | 0.737 | 0.746 | 0.750 | 0.736 | 0.726 |
| 2 | 0.840 | 0.770 | 0.753 | 0.770 | 0.796 | 0.783 | 0.803 | 0.813 | 0.813 | 0.820 |
| 3 | 0.820 | 0.870 | 0.900 | 0.885 | 0.888 | 0.877 | 0.854 | 0.868 | 0.847 | 0.850 |
| 4 | 0.860 | 0.860 | 0.847 | 0.845 | 0.824 | 0.810 | 0.809 | 0.813 | 0.800 | 0.790 |
| 5 | 0.800 | 0.920 | 0.900 | 0.895 | 0.880 | 0.877 | 0.891 | 0.870 | 0.858 | 0.860 |
| 6 | 0.920 | 0.870 | 0.920 | 0.925 | 0.924 | 0.917 | 0.903 | 0.885 | 0.889 | 0.884 |
| 7 | 0.840 | 0.880 | 0.887 | 0.890 | 0.892 | 0.900 | 0.900 | 0.875 | 0.880 | 0.884 |
| 8 | 0.720 | 0.720 | 0.853 | 0.830 | 0.816 | 0.847 | 0.829 | 0.815 | 0.811 | 0.794 |
| 9 | 0.820 | 0.780 | 0.780 | 0.820 | 0.800 | 0.810 | 0.811 | 0.808 | 0.811 | 0.814 |
| 10 | 0.820 | 0.800 | 0.827 | 0.795 | 0.784 | 0.783 | 0.783 | 0.778 | 0.753 | 0.724 |
| 11 | 0.780 | 0.880 | 0.800 | 0.800 | 0.804 | 0.790 | 0.774 | 0.765 | 0.773 | 0.790 |
| 12 | 0.680 | 0.650 | 0.727 | 0.690 | 0.704 | 0.753 | 0.760 | 0.768 | 0.773 | 0.786 |
| 13 | 0.740 | 0.820 | 0.880 | 0.850 | 0.840 | 0.833 | 0.846 | 0.820 | 0.807 | 0.808 |
| 14 | 0.320 | 0.290 | 0.300 | 0.295 | 0.296 | 0.307 | 0.309 | 0.318 | 0.327 | 0.328 |
| 15 | 0.880 | 0.840 | 0.840 | 0.840 | 0.864 | 0.830 | 0.849 | 0.850 | 0.864 | 0.864 |
| 16 | 0.880 | 0.900 | 0.873 | 0.865 | 0.864 | 0.883 | 0.866 | 0.865 | 0.856 | 0.856 |
| 17 | 0.780 | 0.770 | 0.753 | 0.720 | 0.752 | 0.750 | 0.734 | 0.733 | 0.733 | 0.732 |
| 18 | 0.760 | 0.780 | 0.813 | 0.810 | 0.836 | 0.837 | 0.823 | 0.825 | 0.820 | 0.814 |
| 19 | 0.440 | 0.410 | 0.440 | 0.465 | 0.480 | 0.467 | 0.477 | 0.463 | 0.476 | 0.472 |
| 20 | 0.480 | 0.580 | 0.580 | 0.605 | 0.592 | 0.600 | 0.606 | 0.610 | 0.633 | 0.618 |
